# Developing a vaccine against velogenic sub-genotype seven of Newcastle disease virus based on virus-like particles

**DOI:** 10.1186/s13568-023-01617-9

**Published:** 2023-10-17

**Authors:** Masoumeh Firouzamandi, Javad Ashrafi Helan, Hassan Moeini, Alireza Soleimanian, Saeed Khatemeh, Seyed Davoud Hosseini

**Affiliations:** 1 Department of Pathobiology, Faculty of Veterinary Medicine, University of Tabriz, Tabriz, Iran; 2 Institute of Virology, Technical University of Munich, Munich, Germany; 3 Department of Biology, Faculty of Natural Sciences, University of Tabriz, Tabriz, Iran; 4 Razi Vaccine & Serum Research Institute, Arak, Iran

**Keywords:** NDV-VLPs, T-cell response, B-cell response

## Abstract

In the present study, for the first time, we released and assembled the particles of three major structural proteins of velogenic NDV (M, HN, and F glycoproteins) as a NDV-VLPs. The ElISA result of the cytokines of splenocyte suspension cells showed that IL2, IL10, TNF-α, and IFN- ˠ titers were significantly higher (p ≤ 0.05) in mice that were immunized only with NDV-VLPs three times with a 10-day interval, in comparison to those that were immunized with NDV-VLPs twice in a 10-day interval and received a B1 live vaccine boost on the third interval. Flow cytometry results showed that CD8 + titers in the group that only received NDV-VLP was higher than other group. However, serum ELISA results did not show a significantly (p ≥ 0.05) higher NDV antibody titer in NDV-VLPs immunized mice compared to the boosted group. Besides, HI results of SPF chickens vaccinated with NDV-VLPs and boosted with B1 live vaccine were significantly (p ≤ 0.05) higher than those that only received NDV-VLPs. Interestingly, after challenging with NDV sub-genotype VII, all the chickens that were solely vaccinated with NDV-VLPs remained alive (six out of six), whereas two out of six chickens that were vaccinated with NDV-VLPs and also received the B1 live vaccine boost died. In conclusion, our results strongly indicated that the T-cell immune response in an NDV host is more important than the B-cell response. Also, the results of the present study revealed that to completely protect chickens against velogenic NDV strains, a vaccine comprising specific epitopes of velogenic strain is needed.

## Introduction

The term virus-like particles (VLPs) refers to a biocompatible and uncontagious recombinant product with a rod-shaped or icosahedral structure (Pushko et al. 2013; Nooraei et al. 2021). One of the most important infectious diseases that affect the poultry industry — particularly waterfowls like ducks, geese (which have an intermediate function in the viral cross-species transmission), and chickens (more than waterfowls) — is called Newcastle disease (ND), which is caused by Newcastle disease virus (NDV) (Bashir Bello et al. 2020; Guo et al. 2021; Malik et al. 2021). This kind of virus belongs to the genus Avulavirus in the family Paramyxoviridae. Its six critical structural proteins — nucleoprotein, phosphoprotein, matrix protein, fusion protein, hemagglutinin-neuraminidase, and large protein (Qian et al. 2017a) — are encoded by a single strand, negative-sense, and non-segmented RNA genome with six genes 3ʹ-NP-P-M-F-HN-L-5ʹ of NDVs, respectively. RNA editing of the P gene produces two nonstructural proteins — V and W. (Steward et al. 1993; Ferreira et al. 2021) HN and F glycoproteins are the significant protective antigens on the virion surface. The virulence of NDV is related to the F protein, because viral fusion depends on this protein. However, the HN protein helps the F protein function. (Ganar et al. 2014; Wu et al. 2019) NDV is organized into two classes based on the amino acid sequences of the HN and F proteins. Class I has one genotype (genotype I), and class II includes eighteen genotypes (genotype I-XVIII). (Snoeck et al. 2013; Miller et al. 2015; Xu et al. 2019)

Various viral structural proteins are used to produce VLPs, but the critical point in this process is to create a VLP without the viral genome (Qian et al. 2017c) that can still be detected by the immune system (Silva et al. 2017) and stimulate it. Owing to the cavities in their structure, VLPs can be used as carriers for bio and nanomaterials such as imaging substances, drugs, and vaccines. (Chung et al. 2020) Meanwhile, although inactivated and attenuated vaccines have been used to create immunity against viral diseases, they may sometimes lead to various diseases as they revert to virulent forms. (Sarkar et al. 2019) Therefore, designed VLPs are safer than traditional vaccines (Jennings and Bachmann 2008), especially for RNA viruses (Wu et al. 2019) and subunit vaccines, which have a poor immunogenicity. (Mohsen et al. 2017; Brisse et al. 2020; Tariq et al. 2022) VLPs have a crucial role in activating T- and B-cells and antibody responses, which is based on their size, geometry, and viral protein auto-assembling process. (Tornesello et al. 2022) The production of VLPs consists of three main steps: Lysing the cells, eliminating cell debris and large aggregates, and concentration. The final step — concentration — can be achieved through various methods such as high-performance liquid chromatography (HPLC), tangential flow filtration, size exclusion chromatography, and sucrose or cesium chloride density gradient ultracentrifugation. (Tornesello et al. 2022) Due to some post-translational modifications — such as glycosylation and phosphorylation — the source system for VLPs production is crucial. (Zepeda-Cervantes et al. 2020) VLPs can affect the immune system and stimulate both cellular and humoral immune responses. Furthermore, the immune stimulatory capacity of dendritic cells (DCs) — the most potent antigen-presenting cells — is enhanced by VLPs and results in the presentation of viral antigens on MHC I and MHC II. (Quan et al. 2016) Afterward, the activated DCs relocate to the lymph nodes to activate CD4 + T-helper cells (contributing to the activation of Th1, Th2, and CD8 + cytotoxic T-cells). This occurs through a cross-presentation pathway, in which the cells become cytotoxic against chronically infected cells that have presented the MHC-I complex peptide. (Zepeda-Cervantes et al. 2020; Keikha et al. 2021; Murphy and Murphy 2021)

Various components have been used to produce Newcastle disease virus-like particles (NDV VLPs), such as surface glycoproteins, nucleoproteins, and matrix proteins. These components are similar to the wild-type virus in size and shape. In addition, antigen-presenting cells (APCs) like DCs, could take up and process this type of VLP. (Qian et al. 2017c) DC maturation can be activated via NDV VLPs by improving the expression of molecules such as CD40, CD80, CD86, and MHC II on the surface of DCs and triggering the production of cytokines IL-12p70, IL-6, TNF-α, and IFN-γ.

Therefore, NDV VLP is a promising strategy for controlling NDV in poultry species. (Park et al. 2014) Transiently transfected cells with cDNAs that encode the F, M, and HN proteins can prepare NDV VLPs to retain their attachment feature and fusion activities. Another area where NDV VLPs can be used is in assembling and encoding foreign sequences and proteins, because the TM and CT domains of the NDV HN or F proteins are appropriate sites for foreign sequence fusion. NDV VLPs can be used as a novel platform for assembling foreign sequences of other pathogens too. (Qian et al. 2017a) M protein is another membrane-bound particle that is necessary for particle release, and its simultaneous expression with HN and F proteins is vital for effectively incorporating glycoprotein into particles. Therefore, M protein is sufficient and vital for releasing particles, and this protein — along with other proteins such as F and HN — is necessary for incorporating other proteins in particles. (Pantua et al. 2006)

Therefore, the first aim of this study was to prepare NDV-VLPs based on the F, HN, and M proteins of NDV velogenic stain (Beh) sub-genotype VII. The second aim was to evaluate the T-cell and B-cell response of immunized SPF mice and chickens with NDV-VLPs against the velogenic Newcastle disease virus.

## Materials and methods

### Cell culture and vector

Spodoptera frugiperda (Sf-9) insect cells (Invitrogen, USA) were maintained as a suspension culture using Sf-9 serum-free medium (Gibco, USA), supplemented with 10% heat-inactivated Fetal Bovine Serum (FBS) (Gibco, USA) and cultured at 27 °C while stirring at a speed of 130 rpm for baculovirus production. The pFast-Bac expression plasmid (Gibco, USA) was used as a recombinant donor vector to the Bac-to-Bac system.

### Virus and construction of the recombinant pfast-bac donor plasmid

The F, HN, and M gene fragments (NCBI:GenBank: MF417546.1) were amplified by PCR from cRNA isolated from the Newcastle disease virus/Beh strain sub-genotype VII. The M gene was cloned in the reverse direction between the *Kpn*I and *Xho*I sites, under the control of the p10 promoter in the pFast-Bac-Dual plasmid. Also, the reverse direction of the HN gene was cloned after a self-cleaving peptide (T2A) fragment between the *Sal*I and *Xba*I sites, under the control of the p10 promoter. Then, a green fluorescent protein (GFP) sequence (771 bp) was cloned in the forward direction, under the control of the second promoter (pPH) in pFastBac-dual. The F gene was cloned after a self-cleaving peptide (P2A) fragment in the forward direction between the *Neh*I and *Kpn*I sites, under the control of the pPH promoter.

### Transformation of pFast/GFP/F/HN/M to the Bac-to-bac system

The amount of 74 ng of the constructed pFast/GFP/F/M/HN was transformed to 100 µl of DH10 Bac competent cells (Gibco, USA) containing Bacmid for generated recombinant Bacmid by CaCl_2_transformation method. Then, 100 µl of the transformation product was cultured on LB plates containing 50 µg/ ml kanamaycin, 7 µg/ ml gentamicin, 10 µg/ ml tetracycline, 100 µl of Bluo-gal, and 40 ug/ ml IPTG, and incubated at 37 °C for 24–48 h. 10–20 white colonies were harvested and cultured in 6 ml LB containing 6 µl kanamycin, 12 µl tetracycline, and 2.1 µl gentamicin, and incubated at 37 °C for 24–48 h. Finally, the recombinant Bacmid was extracted by Mini-prep of high molecular weight DNA extraction kit (Gibco, USA), and sequenced by specific gene primers and universal M13 primers.

### Transfection of the recombinant bacmid for NDV-VLP production

The recombinant extracted Bacmid was transfected into Sf-9 cells, which were cultured (2_˟_10^6^ cells/ ml with 95% viability) in a T-25 flask with Grace’s insect basal medium (Gibco, USA), supplemented with 10% FBS (Gibco, USA). The flask also contained the following: 10 µl of transfection Expifectamine Sf™ reagent in 500 µl l opti-media that was mixed with 20 µl of recombinant Bacmid in 500 µl opti-media and kept at room temperature for 15 min. The mixed solution (1000 µl) was treated on the cultured Sf-9 cells. Transfected cells were incubated at 27 °C for two to three days to observe the GFP by inverted fluorescent microscope with recombinant baculovirus particles production.

### Recombinant baculoviruses isolation

The budded virus was released into the medium 72–96 h after the transfection (virus in passage P0). The medium containing the virus (P0) was transferred to sterile 15 ml snap-cap tubes and centrifuged at 500 xg for 5 min to remove the cells and large debris. Finally, the supernatant was transferred to a new tube and kept at 4 °C as a P0 virus stock. The isolated virus was propagated by infecting cultured Sf-9 cells, and incubated at 27 °C with 125 rpm for two to three days to produce a large recombinant virus.

### NDV-VLPs purification by sucrose gradient centrifugation

The recombinant virus (P1) released NDV-VLPs in the supernatants. The protein in the supernatants was extracted using I-PER™ Insect Cell Protein Extraction Reagent (Thermo Fisher, Germany) based on the manufacturer’s instructions. The extracted proteins underwent sucrose gradient centrifugation (sucrose 20% and 65%) at 32,000 rpm for 5 h to isolate and purify the NDV-VLPs from large-sized cell debris. Subsequently, purified NDV-VLP was placed in NaCl solutions in dialysis falcon (0.1 N of NaCl in PBS) at 4 °C overnight at 200–300 rpm. The final purification of NDV-VLPs was conducted by centrifuging the liquid at 7000 rpm for 30–60 min in specific filtered falcon tubes (Amicon Ultra, Centrifugal Filters).

### Protein (NDV-VLP) assay by ELISA

The amount of 5 µl of the condensed and purified NDV-VLPs was diluted with 200 µl PBS (phosphate-buffered saline), coated on a 96-well ELISA plate, and incubated at 37 °C for 30 min. The density of the proteins was measured using Pierce™ BCA Protein assay kit (Thermo Fisher Scientific, Germany) according to the following protocol: 200 µl of reagent A was mixed with 4 µl of reagent B, and 200 µl of the mixed solution was added on the coated 96-wells. Finally, the plate was read at 450 nm by an ELISA reader.

### Immuno blotting or Western blotting

The Sf-9 cells were transfected with the recombinant baculoviruses and 48 h after cell transfection proteins lysed using pre-cold 1x RIPA Buffer (Thermo Fisher Scientific, Germany) based on the manufacturer’s protocol. The proteins were incubated on ice for 30 min and run on the SDS-PAGE. Western blotting was done to confirm the presence of the M, HN, and F proteins. Briefly, the SDS-PAGE (MES-based running buffer) gel was transferred to a PVDF membrane by assembling the anode to the cathode of 3 layers of 3MM whatman filter papers, PVDF membrane, SDS gel, and 3 layers of 3MM whatman papers, and ran at 65 MA, 15 V for 1 h. The membrane was incubated in 10 ml blocking solution, which consisted of 10% (v/w) Milk Diluent (KPL, USA) in Tris-buffered saline (TBS) buffer, and gently agitated for 1 h at RT (room temperature). Afterward, the membrane was washed (×3) in 10 ml TBS buffer for 10 min with gentle agitation, transferred to a tray containing chicken anti-NDV polyclonal antibody (Abcam, USA) as the primary antibody (dilutions 1:100 in TBS buffer pH 7.6), and incubated at RT for 1 h or at 4^o^C overnight. On the next day, the membrane was washed (×3) in 20 ml TBS-T (0.01% (v/v) Tween-20 in PBS) for 10 min with gentle agitation. At this stage, goat polyclonal to chicken Ig Y- H&L AP-conjugated (Abcam, USA) antibody was diluted to 1:1000 in 10 ml dilution buffer (TBS buffer) and added to the membrane, followed by at least 2 h of incubation at RT with gentle agitation. The solution complex was washed (×3) with TBST for 5 min, after which a fresh substrate solution — containing 66 µl of the NBT stock (Promega, USA) and 33 µl of the BCIP stock (Promega, USA) mixed in 10 ml alkaline phosphatase buffer — was added to the blots and incubated at RT with gentle agitation. The mixture was watched for color development (5–10 min). The color development was stopped by washing the membrane in distilled water for 10 min. Finally, the membrane was air-dried on filter paper, scanned, and the molecular weight of the proteins was estimated using a protein marker (abm, USA).

### Mice trail

Eighteen female mice, 7–8 weeks of age, were purchased from the Biological Products of Uremia University. After a week of adaptation, the mice were randomly divided into three groups: Group (1) immunized with 50 µl of DNV-VLP in incomplete Freund’s adjuvant at three 10-day intervals; Group (2) immunized with 50 µl of DNV-VLP in incomplete Freund’s adjuvant at two 10-day intervals, on the third interval this group was immunized with 30 µl B1 LaSota strain (Lentogenic) vaccine; and Group (3) immunized with 50 µl PBS as the control group. Two weeks post-immunization, all mice were euthanized by chloroform and their spleens were removed and kept in RPMI medium for T-cells response assay experimental. Blood was collected from the heart for ELISA assay.

### Isolation of spleen lymphocytes

Spleen lymphocytes were isolated based on the protocol used at the virology laboratory of the Technical University of Munich, as follows: The spleens were collected in 15 ml falcons that contained 3–5 ml RPMI on ice and placed a BD cell strainer to 50 ml Falcon. The spleen was smashed and the cell strainer was washed with 20 ml RPMI and then spun down at 1500 rpm, 5 min, and 4 °C. For RBC lysis, the suspended cell pellets were quickly pipetted up and down in 2 ml of ACK buffer (NH4Cl; 8.26 g, KHCO3; 1 g, and Na_2_EDTA; 37.2 mg dissolved in 1000 ml dH_2_O) and then incubated at RT for 1–2 min. The volume was then adjusted to 45 ml with RPMI and placed on ice again. The cells were spun down at 1500 rpm for 5 min at 4 °C, before being resuspended in 2–3 ml RPMI with 10% FBS and filtered again using the cell strainer.

### T-cell stimulation

50 µl of pooled peptides or stimulator protein in RPMI-10 (final con: 2 µg/ ml) was added to each well of a 96-well flat plate. The peptide pool or stimulator protein was prepared as follows: 10 µl of 1 mg/ml PBS peptide or protein 1 ml RPMI-10 was used for T-cell stimulation. 200 µl of cells were collected from each spleen (2 × 10^6^ per well) and added to each well, and the plates were incubated for 24 h at 37^°^C. The supernatant was collected for cytokines ELISA-based assay of IFN-ˠ, IL-2, IL-10, and TNF-α (tumor necrosis factor), and the cells were checked for the stimulation of CD4^+^ and CD8^+^ cells by FACs.

### Measurement of cytokines and NDV-antibody by ELISA

ELISA was carried out to measure the cytokines and assess the B-cells from the mice serums, as explained next: A 96-well flat plate was coated with 100 µl of 2 µg/ml protein (anti-cytokines capture antibodies or NDV antigen) in PBS. The plates were sealed and incubated overnight at 4 °C. The protein was poured into the plates, after which the wells were washed (×5) with PBS/Tween (PBS-T). Afterward, blocked for 2 h using 200 µl of 5% milk powder in PBS. The wells were washed (×3) with PBS-T. Subsequently, the wells were treated with 100 µl of diluted (1:300 in PBS) serum samples for 2 h, and the wells were washed (×5) with PBS-T. The cells were then treated with 100 µl of diluted (1:2000 in PBS) horseradish peroxidase (HRP) goat anti-mouse antibody for 2 h, and the wells were washed (×6) with PBS-T. Finally, 100 µl TMB substrate was added and the mixture was incubated in the dark for 5–10 min. The reaction was stopped by adding 100 µl of stop solution.

### Cell staining for FACs

Single-cell suspensions were diluted to 10 × 10^6 cells/ml in complete DMEM — 100 µl of cells was added to each well. The plate was spun up at 800 x *g* for 3 min at 4 °C, washed three times with cold PBS, and resuspended in 100 µl Fc block (recommended dilution: 1:1000 in FACS buffer). The mixture was incubated on ice for 10 min, spun, and resuspended in 100 µl surface antibody mixture (recommend dilution: 1:200 in FACS buffer). In the next step, it was incubated on ice for 20 min in the dark, pinned, washed once with cold PBS, and resuspended in 200 µl of either BD Cytofix/CytoPerm solution or 1X eBioscience permeabilization solution. The solution was then incubated on ice for 30 min in the dark, pinned 1500 x *g* for 5 min at 4 °C, washed once with 200 µl of either BD Perm/Wash buffer or 1X eBioscience permeabilization buffer, and spun as explained in the previous step. The mixture was resuspended in 100 µl of cytokine stain (recommended dilution: 1:100 in 1X Perm/Wash or permeabilization buffer), incubated on ice for 30 min in the dark, spun as explained in the previous step, washed twice with BD Perm/Wash or eBioscience permeabilization buffer, and spun as described in the previous step. Finally, the cells were resuspended in 100-200bµl FACS buffer and transferred to round-bottom falcon tubes for acquisition on a flow cytometer.

### Chicken immunization and challenge trails

Eighteen 10-day old SPF chicks were randomly divided into three groups, six chicks in each group: Group (1) immunized with 50 µl (~ 32 µg) of DNV-VLP in incomplete Freund’s adjuvant, three times a day with ten day intervals (10, 20, and 30 days old); Group (2) immunized with 50 µl of DNV-VLP in incomplete Freund’s adjuvant two times a day with 10-day intervals, on the third interval, subjects in this group received 30 ul of B1 LaSota strain (Lentogenic) vaccine; and Group (3) the control group, immunized with 50 µl of PBS. Blood samples were collected before each immunization for serological experiments. Two weeks after the third vaccination, each subject was challenged with 50 µl per each eye drops of Newcastle disease virus (10^6.5^ EID50/0.1 ml), velogenic strain (Beh) sub-genotype VII.

### Statistical analysis

All the experiments were done in triplicate. The results have been presented as mean ± standard deviation of the mean (STDEV). The ‎experimental data were analyzed by two-way analysis of variance (ANOVA) using SPSS (IBM Corp. NY, USA).

## Results

### Recombinant pfast-bac donor plasmid construction

**Fig. 1:**
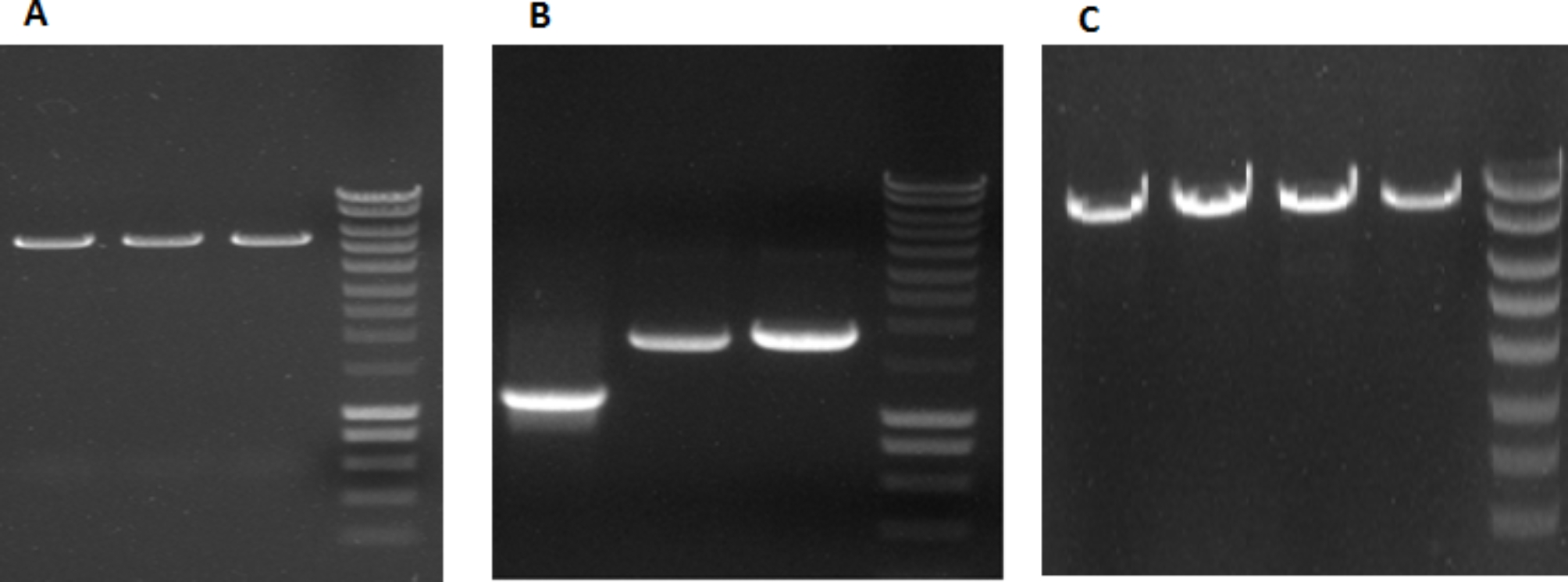
**A**) Enzyme digested products of shuttle vector pFastBac Dual (5238 bp) before cloning on 1% agarose gel. DNA ‎ladder 200 bp-10 kb (200, 400, 600, 800, **1000, 1500**, 2000, 2500, 3000, 4000, 5000, 6000, **8000, 10,000**; Thermo Science fisher, ‎Germany).‎ **B)** PCR products of four structural genes of NDV 2018‎‏/‏‎(VIId) Ck/IR/MAM strain on 1% agarose gel; M gene (1094 ‎bp), F gene (1661 bp), and HN gene (1716 bp), respectively, DNA ladder 200 bp-10 kb. **C**) Enzyme digested products of pFast (5238 bp) + GFP (717 bp) + P2A (54 bp) + F gene (1661 bp) + M gene (1094 bp) = 8764 ‎bp for cloning verification on 1% agarose gel; DNA ladder 200 bp-10 kb (Thermo Sciencefisher, Germany)

**Fig. 2:**
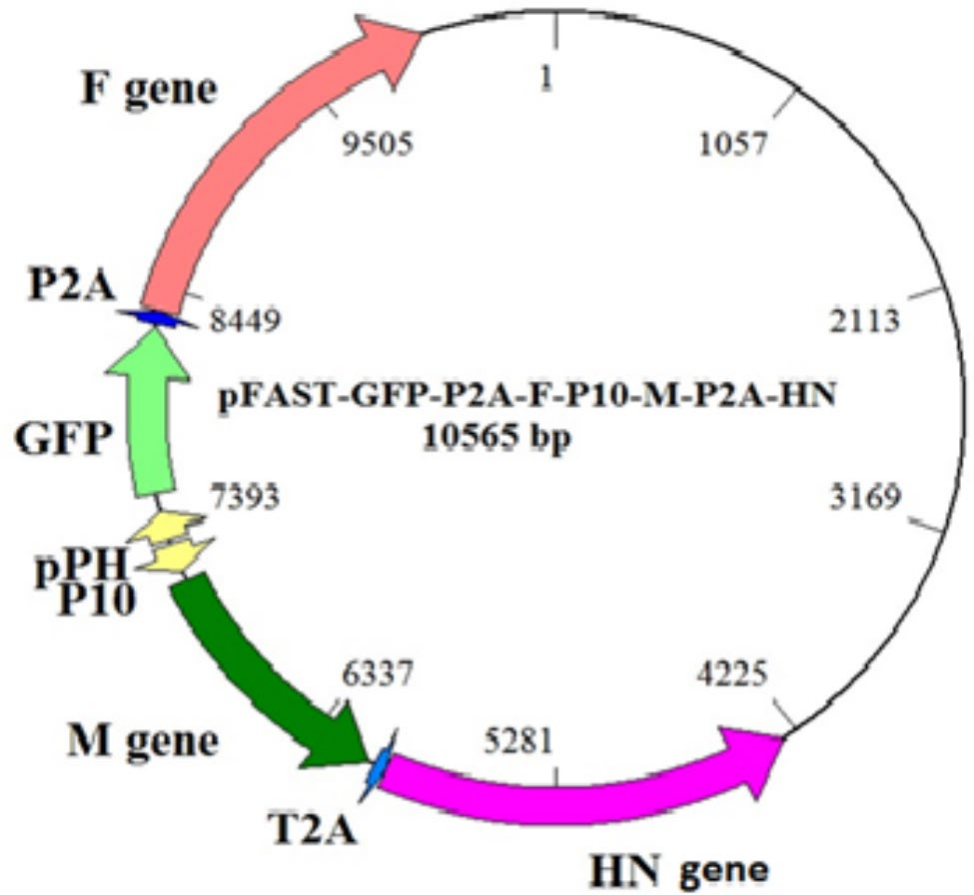
A schematic diagram of constructed pFast/GFP/F/M/HN shuttle vector

The F, HN, and M genes of NDV sub-genotype VII were successfully amplified by specific primers (Fig. 1). Recombinant pFast/GFP/F/M/HN was constructed (Fig. 2).

### Transformation of pFast/GFP/F/HN/M to the Bac-to-bac system

We had to examine whether pFast/GFP/F/HN/M had transformed to the Bac-to-Bac system, and whether the pFast/GFP/F/HN/M shuttle vector had been introduced to the bacterial recombination system (which produces the Bacmid containing our interested genes). To do so, we observed the PCR product (2300 bp) — which was produced using the forward F gene primer and reverse Bacmid primer — on 1% agarose gel.

### Transfection of the recombinant bacmid for NDV-VLP production

**Fig. 3:**
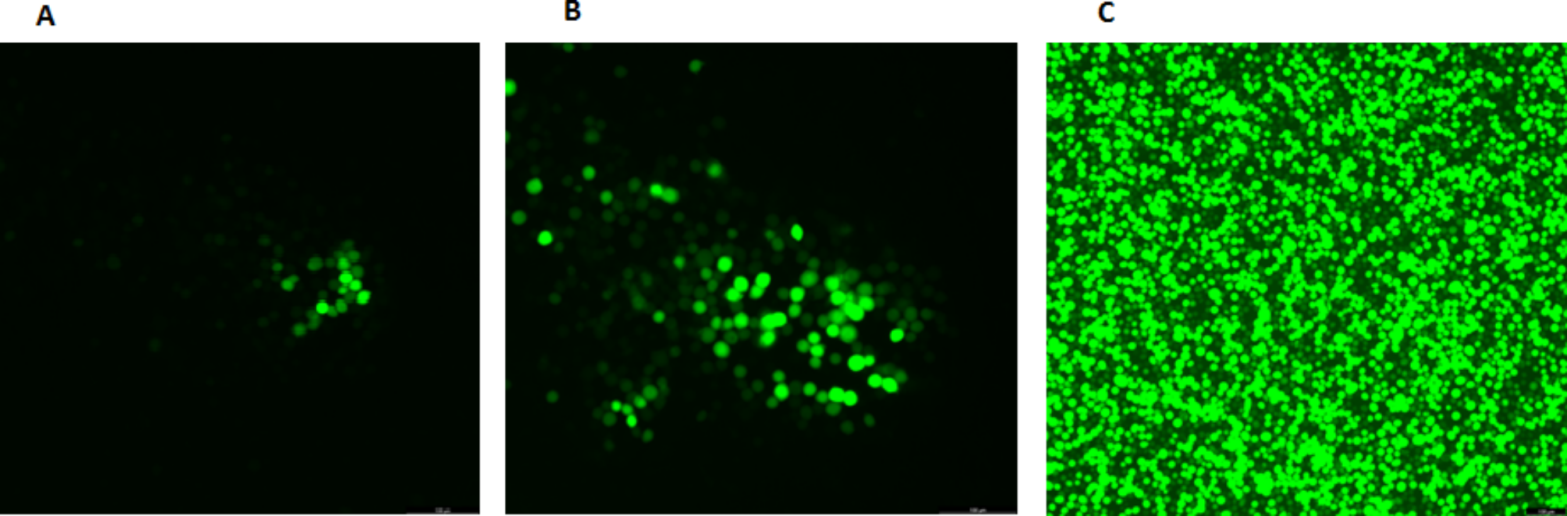
Expression of green fluorescent protein (GPF) in Sf-9 cells in vitro. The Sf-9 cells were transfected with the recombinant baculoviruses (BAC-mid). After 48 h (**A**), 72 h (**B**), and 96 h (**C**) of transfection, successful transfection was confirmed with green fluorescent observation by an inverted fluorescence microscope (magnification, x400)

Green fluorescent protein was observed after 48, 72, and 96 h post-transfection of recombinant Bacmid into Sf-9 cells (Fig. 3) under an inverted fluorescent microscope (Thomas Scientific, USA).

### NDV-VLPs purification by sucrose gradient centrifugation

**Fig. 4:**
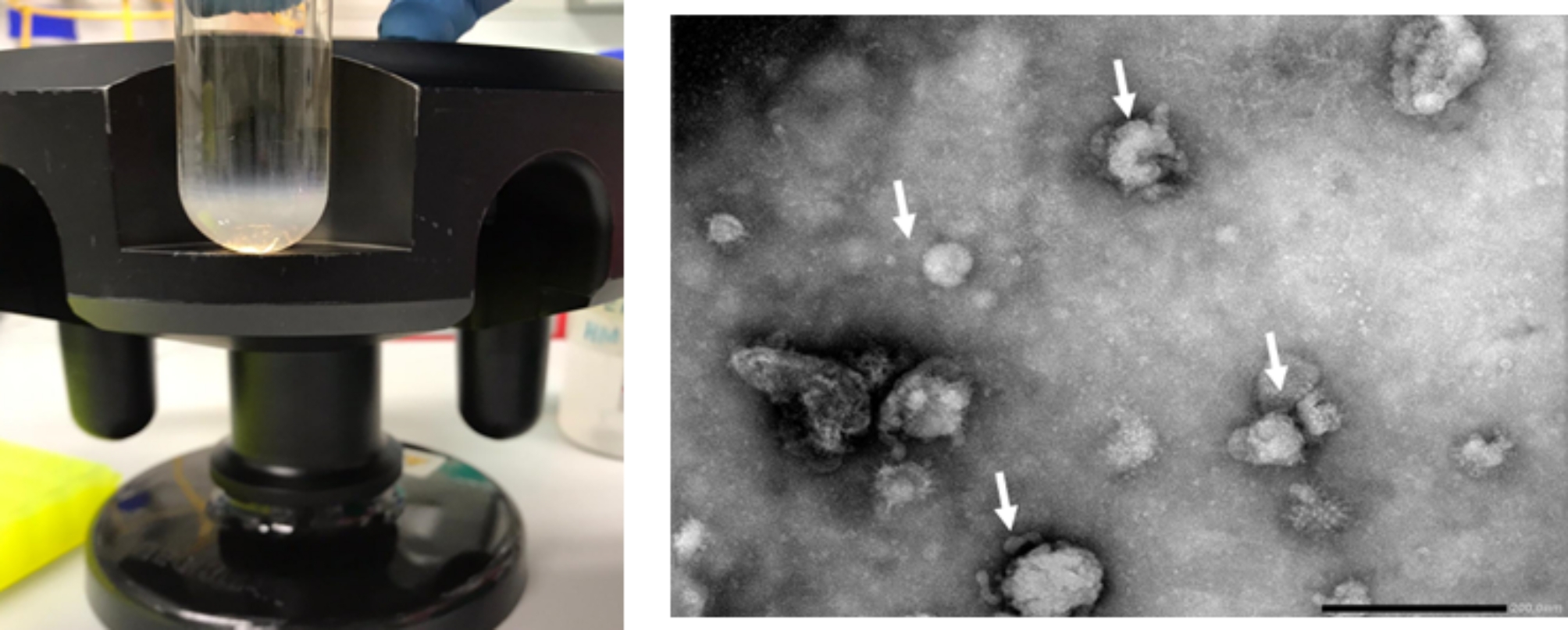
**A**) VLP of Newcastle disease virus (NDV) in sucrose gradient. The Sf-9 cells were transfected with ‎recombined BAC-mid. After 96 h, the supernatant was carried out on 20% and 65% sucrose (centrifuged at 32,000 rpm for 5 ‎h). **B**) Electron microscopy: NDV-VLPs.

The proteins extracted from recombinant baculoviruses were carried out on a sucrose gradient and centrifuged for 5 h at 32,000 rpm. Concentrated and purified NDV-VLPs were observed and collected in 65% sucrose (Fig. 4a). Once the sucrose was removed from the NDV-VLPs by dialysis falcons (NaCl, 0.1 N in PBS), NDV-VLPs concentration was measured as 635 µg/ml by an ELISA reader. Finally, NDV-VLPs were scanned with an electron microscope (Fig. 4b) (Thermo Fisher Scientific. USA).

### Western blotting

**Fig. 5:**
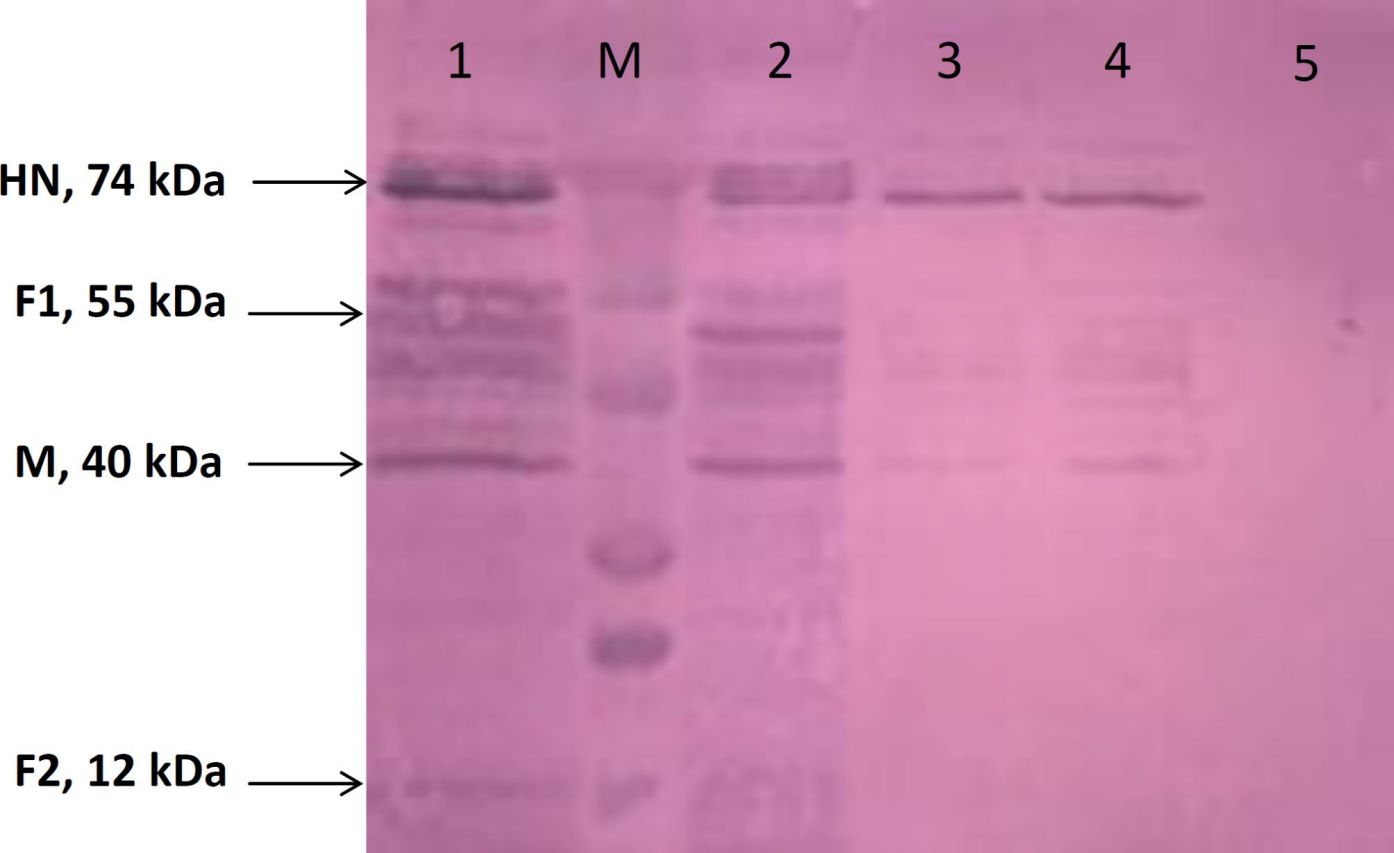
Translation expression analysis of the viral genes in vitro by Western blotting. The Sf-9 cells were transfected with the recombinant baculoviruses. Protein expression was studied 48 h after cell transfection. Molecular masses are in kilodaltons (kDa). Lanes 1 & 2: concentrated VLP samples from Sf-9 cells treated with recombinant baculoviruses containing NDV-HN, F, and M genes; M: Opti-Protein Ultra Marker (abm, USA); Lanes 3&4: diluted VLP samples of Sf-9 cells treated with recombinant baculoviruses containing NDV-HN, F, and M genes; Lane 5: untreated Sf-9 cells as the negative control

Proteins was extracted from the transfected Sf-9 cells by recombinant baculoviruses and carry out for western blotting and a 55 kDa of fusion protein (F1), a 78 kDa of HN protein, a 40 kDa of M protein, and 12 kDa of fusion protein (F2) confirmed by Western blot (Fig. 5).

### Flow cytometry results

**Fig. 6:**
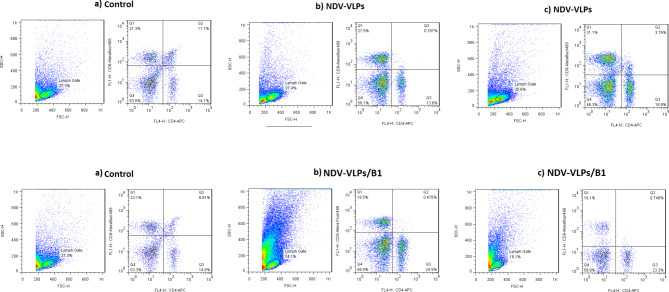
Gating strategy for the flow cytometric analysis of mice T-cells, platelets, and their subpopulations. A single-cell suspension was stained with fluorochrome-conjugated antibodies against CD4-FL4- APC and CD8-FL1-488. Data were collected with a MACSQuant flow cytometer and analyzed with FlowJo Software. Lymphocytes were identified by their scatter properties (FSC-A × SSC-A plot) and doublets were excluded by gating on FSC-A × FSC-H. CD4 + or CD8 + cells represent T-cell subsets in immunized mice with (a) only NDV-VLPs and (b) NDV-VLPs boosted with B1 NDV live vaccine

The CD4 + and CD8 + lymphocytes in the splenocyte cells were assayed by a flow cytometer (Fig. 6). Results showed that CD8 + lymphocytes were higher in mice immunized with only NDV-VLPs, in comparison to those immunized with NDV-VLPs and B1 live vaccine. This result implies that immunizing with NDV-VLPs could raise the level of T-cell response by three times due to specific virulent strain NDV epitopes. B1 live vaccine belongs to the LaSota strain (lentogenic), which could not increase the level of T-cell response due to the low interaction of its epitopes with the immune system.

### Cytokines ELISA results of the immunized mice

**Fig. 7:**
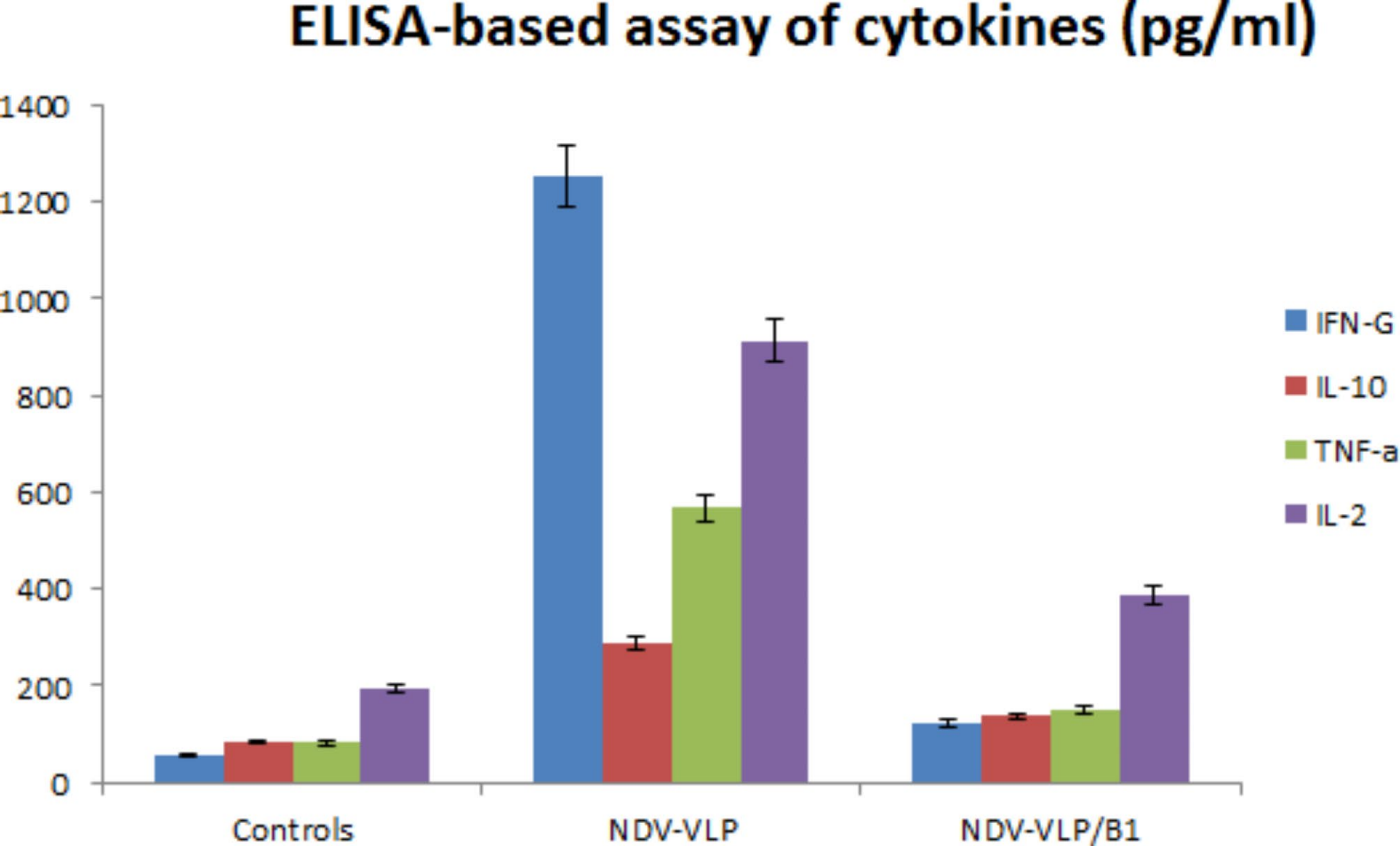
ELISA results of IL2, IL10, TNF-α, and IFN- ˠ specific cytokines from the splenocyte suspension cells of mice immunized with only NDV-VLPs and those immunized with NDV-VPLs and boosted with B1 NDV live vaccine compared to the control group

IL2, IL10, TNF-α, and IFN- ˠ specific cytokines from the splenocyte suspension cells were assayed by ELISA. Interestingly, the highest IFN- ˠ titer was observed in the group immunized with only NDV-VLPs. Moreover, IL2, TNF- α, and IL10 titers were significantly higher in the group immunized with NDV-VLPs compared to the group immunized with NDV-VLPs and B1 live vaccine and the control group. In the group immunized with NDV-VLPs and B1 live vaccine, IL2 titer was significantly higher than other cytokines assayed (Fig. 7).

### Serum ELISA results of the immunized mice

**Fig. 8:**
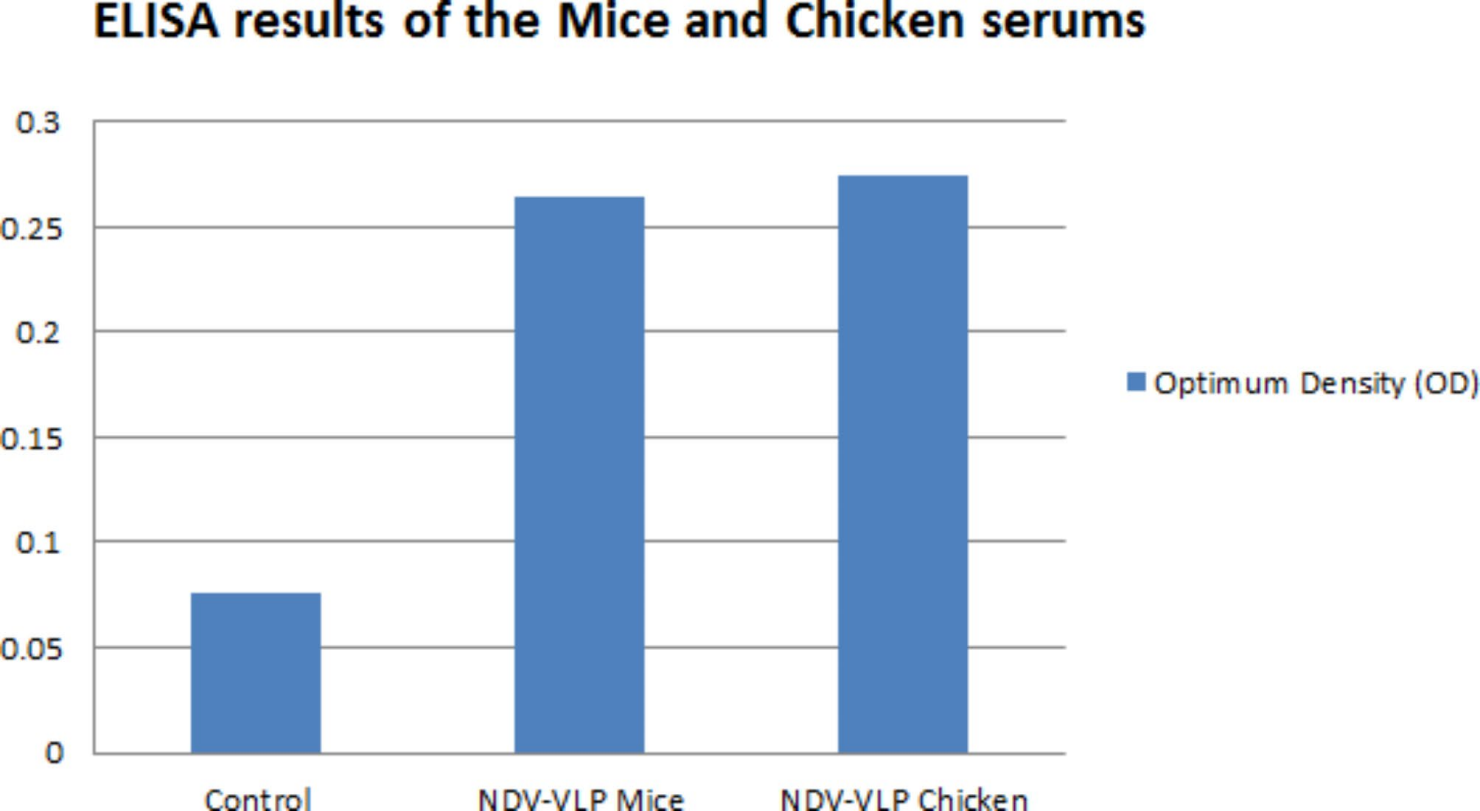
ELISA results of mice serums immunized with only NDV-VLPs and those immunized with NDV-VPLs and boosted with B1 NDV live vaccine compared to the control group

In the group immunized with only NDV-VLPs, NDV antibody responses two weeks after the third immunization did not have a significantly (p ≥ 0.05) higher titer than those immunized with NDV-VLPs and the B1 vaccine. However, both groups showed significantly (p < 0.05) higher titer values compared to the control group (Fig. 8).

### NDV-HN antibody response of immunized chickens

Hemagglutination Inhibition (HI) assay on the serum samples of chickens at 20, 30 days old before vaccination and two-week after third vaccination (before challenge) were developed as the HI antibody titer. HI titers were in the low range at 20 days after the first vaccination (just before the second). In both groups — that is NDV-VLPs group and NDV-VLPs boosted with B1 vaccine group — HI titers increased to log_2_ 5.96 ± 0.73 and 6.69 ± 0.87, respectively, two weeks after the third vaccination (Table 1).

**Table 1:** Mean HN antibody titers assayed by HI test (log_2_) of SPF chickens after immunization with different vaccination programs (geometric mean ± sd)

	Mean HI titers			
Group names	20 days old chicken	30 days old chicken	Two-week post third vaccination	Alive chickens after 6 days post challenge
NDV-VLPs	1.5 ± 0.67	2.1 ± 0.32	5.96 ± 0.73	6out of 6 (100%)
NDV-VLPs boosted with B1 vaccine	1.3 ± 0.56	2.3 ± 0.15	7.89 ± 0.87.5	4 out of 6 (66%)
Control	0.00	0.00	0.00	0 out of 6 (0%)

### Chickens’ challenge results

All subjects were challenged with the velogenic virus sub-genotype VII two weeks after the third vaccination. The chickens showed virulent ND signs and symptoms in the control group two and three days after the challenge — the entire group died on the third day.

However, the group that only received NDV-VLPs showed silent ND signs in their eyes and remained alive after the challenge. In the group immunized with NDV-VLPs and B1 vaccine, two of six chickens showed virulent ND signs from the second day after the challenge and died on the sixth day (Table 1).

## Discussion

Previous studies show VLPs are one of the best methods for preventing and controlling NDV. Therefore, many investigations have been conducted to improve this type of vaccine. The present study showed that NDV-VLPs created as a vaccine by the F, HN, and M NDV proteins can protect chickens against virulent strain sub genotype VII of Newcastle disease. Moreover, we found that the live B1 LaSota strain (Lentogenic) vaccine increased NDV antibodies, but could not completely protect chickens from sub-genotype VII NDV. Our results are in line with Jing et al., who created a package of NDV structural HN and M proteins that used a Bac-to-Bac baculovirus expression system. These VLPs led to a boosted protection against NDV by activating DCs (Jing et al. 2017). (Qian et al. 2017b) This team went on to generate VLPs with NDV M and HN proteins against VII NDV to induce chicken bone marrow-derived dendritic cells (chBMDC) to become mature. This resulted in increased production of protective serum IgG and increase the expression of MHC II, CD86, CD80, and CD40, in addition to cytokine secretions of IL-6, IL-12p70, IFN-c, and TNF-a. (Xu et al. 2020) In another research, Shen et al. used the Bac-to-Bac system to create avian influenza virus-based VLPs comprising NDV proteins. (Shen et al. 2013; Xu et al. 2019) This study uncovered the vital role of NF-κB and TLR4 in DC maturation after being provoked by NDV VLPs.

As for the antigen-presenting ability of migratory DCs, they found that previously transferred allogeneic mDCs can efficiently activate CD4 + and CD8 + T-cells to produce intracellular IFN-γ and IL-4 in response to NDV VLPs. (Xu et al. 2020)

Our results showed that CD8 + titers in the group immunized with NDV-VLPs alone were higher than the group immunized with NDV-VLPs and boosted with B1 live vaccine. To sum up, these results revealed that NDV VLPs promote DC homing and presentation in the primary immune response, resulting in the activation of the naïve T-cell population. (Yoshimura et al. 2001; Qian et al. 2017c)

In another research, vaccination with NDV VLPs showed that T-cell responses were as efficient as an ND virus infection, and comparable amounts of IFN- ɣ ^+^ CD8^+^ cells were found in the spleens of vaccinated animals. (McGinnes et al. 2010) Our cytokines ELISA results of splenocyte suspension cells showed that IL2, IL10, TNF-α, and IFN- ˠ titers were significantly higher (p ≤ 0.05) in the group immunized only with three NDV-VLPs treatments over a period of three 10-day intervals compared to the group vaccinated twice with NDV-VLPs in two 10-day intervals and with a B1 live vaccine on the third. The process of DCs presenting antigens to naïve T-cells needs the assistance of MHC II and co-stimulatory molecules CD80 and CD86. VLPs can up-regulate the surface molecules of mDCs, which is necessary for T-cell activation.

Consequently, this research has indicated that NDV VLPs can activate all CD8 + IL-4+, CD8 + IFN-γ+, CD4 + IL-4+, and CD4 + IFN-γ + T-cells from the VLP group, and the NDV VLP-treated DCs of the spleen can activate specific T-cells. (Qian et al. 2017c) Our results are in line with Qian, J. et al., who demonstrated that novel VLPs can be produced with genotype VII NDV HN and M proteins, induce an IgG1-dominant response, and cause humoral immune responses, since NDV VLP provoked the T-cell immune responses. (Qian et al. 2017a).

## Data Availability

The authors confirm that the data supporting the findings of this study are available within the article and its supplementary materials.

## Abbreviations

VLPs: Virus-Like Particles
NDV: Newcastle Disease Virus
ElISA: Enzyme-Linked Immunosorbent Assay
SPF: Specific-Pathogen-Free
HPLC: High-Performance Liquid Chromatography
DCs: Dendritic Cells
APCs: Antigen-Presenting Cells
Sf-9: Spodoptera frugiperda
FBS: Fetal Bovine Serum
GFP: Green Fluorescent Protein
TBS: Tris-Buffered Saline
HRP: Horseradish Peroxidase
PBS: Phosphate-Buffered Saline
HI: Hemagglutination Inhibition
